# Causal inference of inflammatory proteins in infertility: a Mendelian randomization study

**DOI:** 10.3389/fendo.2025.1448530

**Published:** 2025-02-25

**Authors:** Peng Chen, Sha Ni, Ling Ou-Yang

**Affiliations:** Department of Obstetrics and Gynecology, Shengjing Hospital of China Medical University, Shenyang, Liaoning, China

**Keywords:** infertility, Mendelian randomization, drug target, molecular biomarker, CXCL6

## Abstract

**Background:**

Infertility affects 8-12% of couples globally, manifesting as a complex reproductive disorder with varied causes, negatively impacting emotional, physical, and social well-being. Inflammation is implicated in many diseases, including male and female infertility.

**Methods:**

This study employed Mendelian randomization (MR) with two-sample, bidirectional, and mediation approaches to explore the relationship between circulating inflammatory proteins and infertility. Causal analysis was conducted using inverse variance-weighted (IVW) and MR-Egger regression, supplemented by enrichment analysis, protein-protein interaction (PPI) network exploration, and drug signature analysis.

**Results:**

Our findings identified a significant positive correlation between C-X-C motif chemokine 6 (CXCL6) and male infertility, positioning CXCL6 as a potential therapeutic target or biomarker. No causal links were detected between circulating inflammatory proteins and female infertility post-FDR adjustment. Minor mediation effects were observed for metabolites such as androstenediol monosulfate, arachidonoylcholine, and serum phosphate to glycerol ratio. Cytokine-related pathways emerged as significant in both male and female infertility. Gene-drug interaction analysis highlighted the need for further investigation of pioglitazone in treating female infertility.

**Conclusion:**

This study establishes a potentially causal relationship between CXCL6 and male infertility, suggesting its potential as a drug target or molecular biomarker. The integrative approach combining causal inference with molecular pathway and drug interaction analysis opens new avenues for understanding and treating infertility.

## Introduction

Infertility is a worldwide human health problem that affects approximately over 186 million individuals globally (1, 2). The causes of infertility vary, with female factors accounting for about 70% of infertility cases, with common causes including ovulatory disorders and fallopian tube damage (3). Male factors contribute to the remaining roughly 30% of infertility cases due to causes such as abnormal sperm count, morphology, or lifespan (4). Independent of etiology, infertility has a profound emotional, physical, and social impact on couples and results in a significant psychological burden (5). Thus, understanding the causes of infertility and exploring a possible causal link between risk factors and infertility are crucial for affected couples and healthcare professionals.

Inflammation is a physiologic host response to infection or injury. However, aberrant inflammatory responses result in tissue damage and are central to the pathogenesis of multiple diseases, including reproductive diseases (6). Inflammation can have significant effects on both male and female infertility. For example, untreated sexually transmitted diseases in females can lead to tubal factor infertility due to tubal inflammation, damage, and scarring (7). Mutations in TNFAIP3 have been linked to both infertility and central nervous system inflammation, highlighting the role of inflammation in female infertility (8). In males, inflammation can lead to leukocytospermia, due to the production of reactive oxygen free radicals, and chronic testicular inflammation, such as orchitis, is also known to cause male infertility (9, 10). The role of inflammation and oxidative stress in male infertility is well-documented, with mechanistic pathways linking causative factors of male reproductive tract inflammation, oxidative stress induction, and oxidant-sensitive cellular cascades leading to male infertility (11, 12). The presence of inflammatory states and the identification of semen markers of inflammation are crucial for the diagnostic-therapeutic management of male infertility (13). These results emphasize the critical role of inflammation in both male and female infertility, highlighting the need for further research and therapeutic interventions targeting inflammatory pathways to address infertility.

Inflammatory responses are orchestrated by a complex network of cells and inflammatory mediators, such as circulating inflammatory proteins (CIPs). For instance, high levels of CIPs, like growth-regulated oncogene-alpha (GROα) are related to increased risks of female infertility (14). CXCL6 plays a key role in recruiting neutrophils to facilitate non-specific immunity during the process of inflammation (15). Additionally, GCP-2/CXCL6 exhibits antibacterial activity and plays key roles in host defense of the male urogenital tract as well as during fertilization (16). Furthermore, CIPs are vulnerable to the direct effects of conventional small molecules or biologics, making them attractive potential drug targets (17). Therefore, the discovery of genetic determinants affecting CIP levels may offer important clues about the etiology of multiple diseases, such as infertility.

Randomized controlled trials (RCTs) are a gold standard means to test hypotheses at a population level (18, 19). However, RCTs require intensive human resources, cost, and time, and some interventional strategies are not suitable for RCT-dependent assessments. Recently, Mendelian randomization (MR) has gained attention as a complementary method of exploring the relationship between the treatment and outcome in patients, based on the frequency of single nucleotide polymorphisms (SNPs) as the instrumental variable (IV). This approach might avoid the confounding bias of conventional epidemiological investigations (20). Here, we performed a comprehensive two-sample MR analysis to determine the potential causal association between CIPs and infertility.

## Materials and methods

### Study design

**Figure 1:**
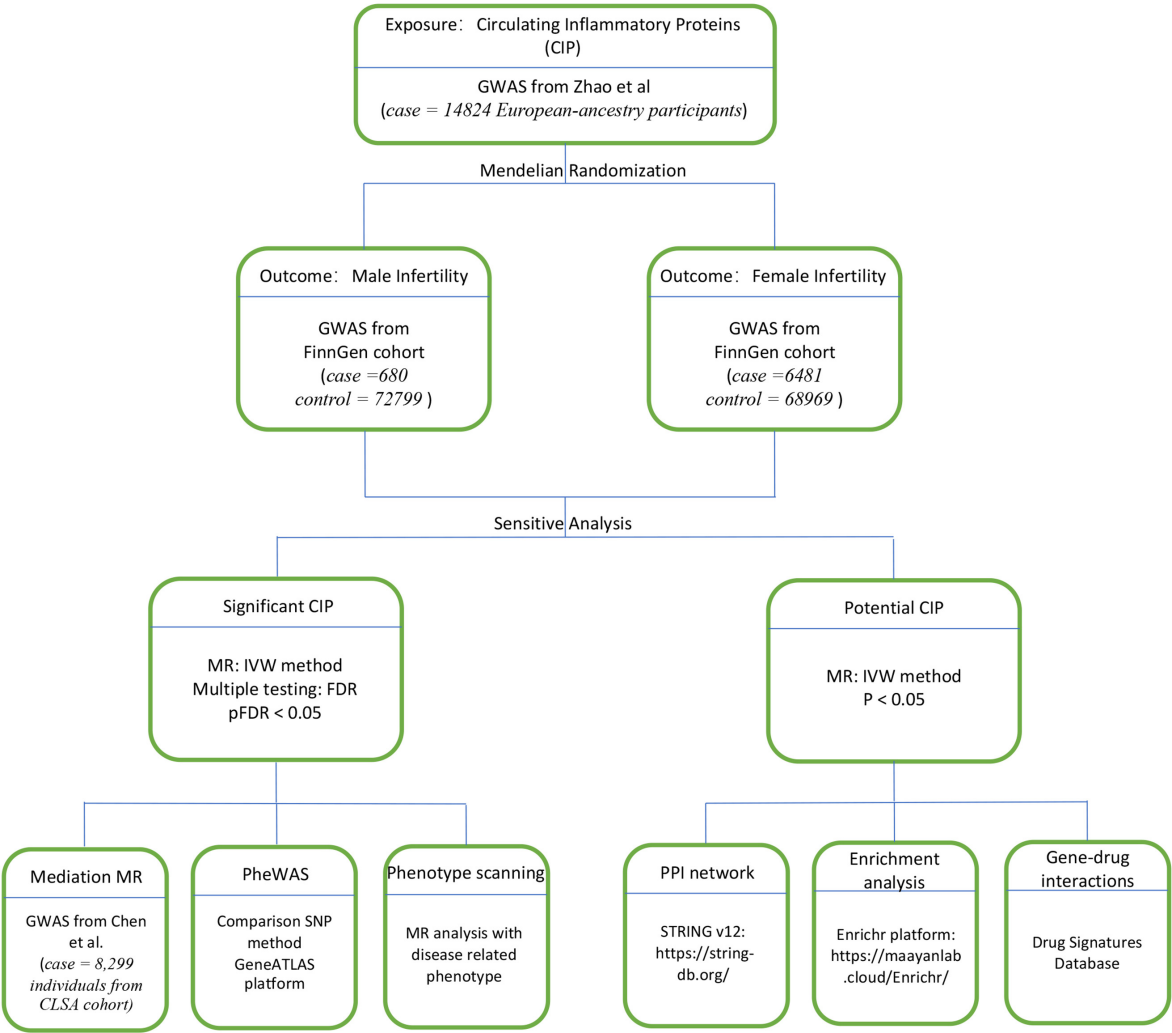
Study design. Overview of the MR study design.

This epidemiologic MR study was carried out by adhering to the STROBE guidelines (21). The reliable implementation of MR is based on three major assumptions: (i) relevance (genetic variants are strongly linked to the exposure), (ii) independence (variants are not correlated with confounders), and (iii) absence of horizontal pleiotropy (variants affect the outcome only via the exposure) (22). Here, we utilized bidirectional and 2-sample MR to understand the pathological correlation between CIPs and infertility. Then we used significant positive CIPs to construct a phenome-wide association study (PheWAS) and phenotype scanning analysis. We also used MR to assess for a casual relationship between significant CIP and plasma metabolites to find potential mediators of infertility. A protein-protein interaction (PPI) map was then generated between the positive CIP and infertility, and we performed functional enrichment analysis and drug signature analysis with positive CIPs (**Figure 1**).

### GWAS summary data sources

The genome-wide pQTL (protein quantitative trait loci) mapping for CIP was obtained from studies by Zhao et al. (23). This dataset contained 91 CIP measured using the Olink Target Inflammation panel in 11 cohorts, with a total of 14,824 participants of European ancestry. The plasma metabolite data was obtained from a series of large GWASs by Chen et al. (24). This dataset included 1,091 metabolites and 309 metabolite ratios in 8,299 individuals from the Canadian Longitudinal Study on Aging (CLSA) cohort. We used publicly available GWAS summary datasets for male infertility from the FinnGen cohort (R9 release, total n = 73479; case =680, control = 72799) and female infertility data from the FinnGen cohort (R9 release, total n = 75450; case =6481, control = 68969). FinnGen is a large public-private collaboration that includes genomic data from 500,000 individuals of Finnish individuals who are over 18 years of age (25). These GWAS summary datasets and the GWAS summary datasets data for sensitivity analysis are listed in **Table 1**.

**Table 1:** Description of the contributing GWAS studies.

Traits	PMID	Case size	Sample size	Ancestry
Exposure				
Circulating inflammatory proteins	37563310	NA	14824	European
Outcomes				
Male infertility	FinnGen cohort	680	73479	European
Female infertility	FinnGen cohort	6481	68969	European
Mediator				
Plasma metabolites	36635386	NA	8299	European
Phenotypes				
Myocardial infarction	33959723	11081	484598	European
Allergic disease asthma hay fever or eczema	29083406	180129	360838	European
Celiac disease	22057235	12041	24269	European and Asian
Colorectal cancer	34594039	14886	637693	European and Asian
Coronary artery disease	29212778	122733	547261	European
Diabetes type 1	34012112	18942	501638	European
Glaucoma primary open angle	FinnGen cohort	4433	214634	European
Hypertension	33959723	129909	484598	European
Hypothyroidism	29892013	NA	473703	European
Inflammatory bowel disease	33959723	4101	480497	European
Juvenile idiopathic arthritis including oligoarticular and rheumatoid factor negative polyarticular JIA	23603761	2816	13056	European
Primary biliary cirrhosis	22961000	2861	11375	European
Primary sclerosing cholangitis	27992413	2871	14890	European
Rheumatoid arthritis	33959723	5427	484598	European
Selective immunoglobulin A deficiency IgAD	27723758	1635	6487	European
Self-reported psoriasis	33959723	5427	484598	European
Systemic lupus erythematosus	34594039	964	659165	European and Asian
Vitiligo	FinnGen cohort	131	207613	European

### SNP selection

Exposure-associated independent SNPs (r^2^ = 0.001, ≥10000 kb) were selected according to their genome-wide (GW) significance (p<1.0×10^−5^) (26). The F-statistic (F-stat) of a phenotype is a measure of instrument strength (IS) and is related to the proportion of variance. An F-stat of at least 10 variables indicates a relatively lower risk of instrument bias (IB) in MR analysis (27). The number of SNPs for each CIP and the corresponding scale unit and F-stats are presented in **Supplementary Table S1**.

### MR analysis

First, SNPs were harmonized to the exposure and outcome in an allele-specific manner to ensure proper alignment of effects. Wherever applicable, if any instrumental SNPs for the exposure were absent in the corresponding outcome dataset, a proxy was incorporated using GVs in the linkage disequilibrium (r^2^ > 0.8).

Initially, we employed an inverse variance-weighted (IVW) meta-analysis approach for MR analysis (28), in addition to the weighted median (29) and MR-Egger regression (MR-ER) approach. Multiple testing was conducted based on the false discovery rate (FDR) method and a pFDR<0.05 was deemed a significant CIP. P values <0.05 indicated a potential CIP.

The MR-ER method was further used to assess any potential impacts of the directional pleiotropy (DP) (30) and to perform the MR-pleiotropy residual sum and outlier (MR-PRESSO) method. A P value > 0.05 indicated no horizontal pleiotropy. Data heterogeneity was determined by Cochran’s Q test (31), with P > 0.05 indicating no heterogeneity Leave-one-out (LOO) analysis indicated the influence of a single SNP on overall estimates.

“TwoSampleMR v0.5.8,” “MRPRESSO v1.0”, and “MendelianRandomizaiton v0.9.0” packages in R v4.3.1 (source codes: https://github.com/studentyaoshi/MR) were applied for statistical analyses.

### Reverse causality detection

To detect potential RC, genetic instruments for infertility were selected in a similar strategy to CIPs selection from GWAS datasets for bidirectional MR analysis (32). The effect was estimated by MR-Egger, IVW, and weighted mode. A P<0.05 indicated statistical significance.

### Mediation MR analysis

In mediation terms, a two-step MR was applied to distinguish between indirect and direct effects. Significant CIP-related genetic instruments (GIs) were used to assess the causal effects of plasma metabolites. GIs for plasma metabolites were then used to determine the effect of each plasma metabolite on possible CIP-related infertility. Notably, if significant CIP-related infertility was found to influence the mediator, we used the “product of coefficients” approach to evaluate the indirect effects of significant CIPs on infertility. The delta method was applied to calculate the standard error (SE) for an indirect effect.

### PheWAS

To evaluate the horizontal pleiotropy of significant CIPs, a PheWAS was performed. We compiled a comparison SNP-set to serve as a control for PheWAS analyses. Four control SNPs were matched to the significant CIP-associated SNPs on: minor allele frequency (± 5%), surrounding gene density (± 50%), distance to the nearest gene (± 50%), and as a proxy for haplotype block size, the number of other SNPs in LD at R 2 ≥ 0.50 (± 50%) (33). GeneATLAS is a searchable dataset for 778 traits (118 quantitative, 660 binary) and associations with 9,113,133 genetic variants (genotyped or imputed) and can be queried for genetic or phenotypic data to assess genotype-phenotype associations (34). We queried GeneATLAS for trait associations with significant CIPs. Nominally significant SNP-trait associations (p < 0.01) were carried forward to trait-enrichment analyses, as previously described (35). PheWAS associations of the significant CIP SNPs with 778 phenotypic traits were compared to PheWAS results for the control SNP-set. We used Fisher’s exact tests to compare the frequency at which individual traits were associated with significant CIPs SNPs versus control SNPs to determine if traits were enriched for association with significant CIP risk variants. Fisher’s exact p-values for trait enrichment underwent FDR correction to adjust for multiple testing.

### Phenotype scanning

Phenotype scanning was carried out by searching the previous GWAS via a ‘phenoscanner’ to reveal the associations of an identified CIPs with other traits (36). A pleiotropic SNP was considered to possess: (i) a significant genome-wide association (P<5 × 10^−8^), (ii) a relationship with European descendants in GWAS, and (iii) associations with any known etiological risk factors, including protein, metabolic, and clinical traits. Additionally, the LD r^2^ among identified CIPs revealed their potential linkages with potential infertility drug targets.

### PPI network

The PPI network analysis (STRING v12; https://string-db.org/; minimum interaction score = 0.4) was used to detect any associations between positive CIPs.

### Functional enrichment analysis of positive CIPs and gene-drug interactions

Enrichr platform (https://maayanlab.cloud/Enrichr/), an independent web server for gene set enrichment analysis (37), was used to perform functional enrichment analysis on positive CIPs. Drug Signatures Database (DSigDB) was used to identify drug molecules that interacted with positive CIPs through the Enrichr platform.

### Data availability

All relevant data for this study are included in the article or uploaded as online supplemental information. Full GWAS summary statistics for the exposure and outcome data used in the manuscript can be found at https://www.ebi.ac.uk/gwas, https://gwas.mrcieu.ac.uk/, or https://r9.risteys.finngen.fi.

## Results

### Associations between CIPs and male infertility

**Figure 2:**
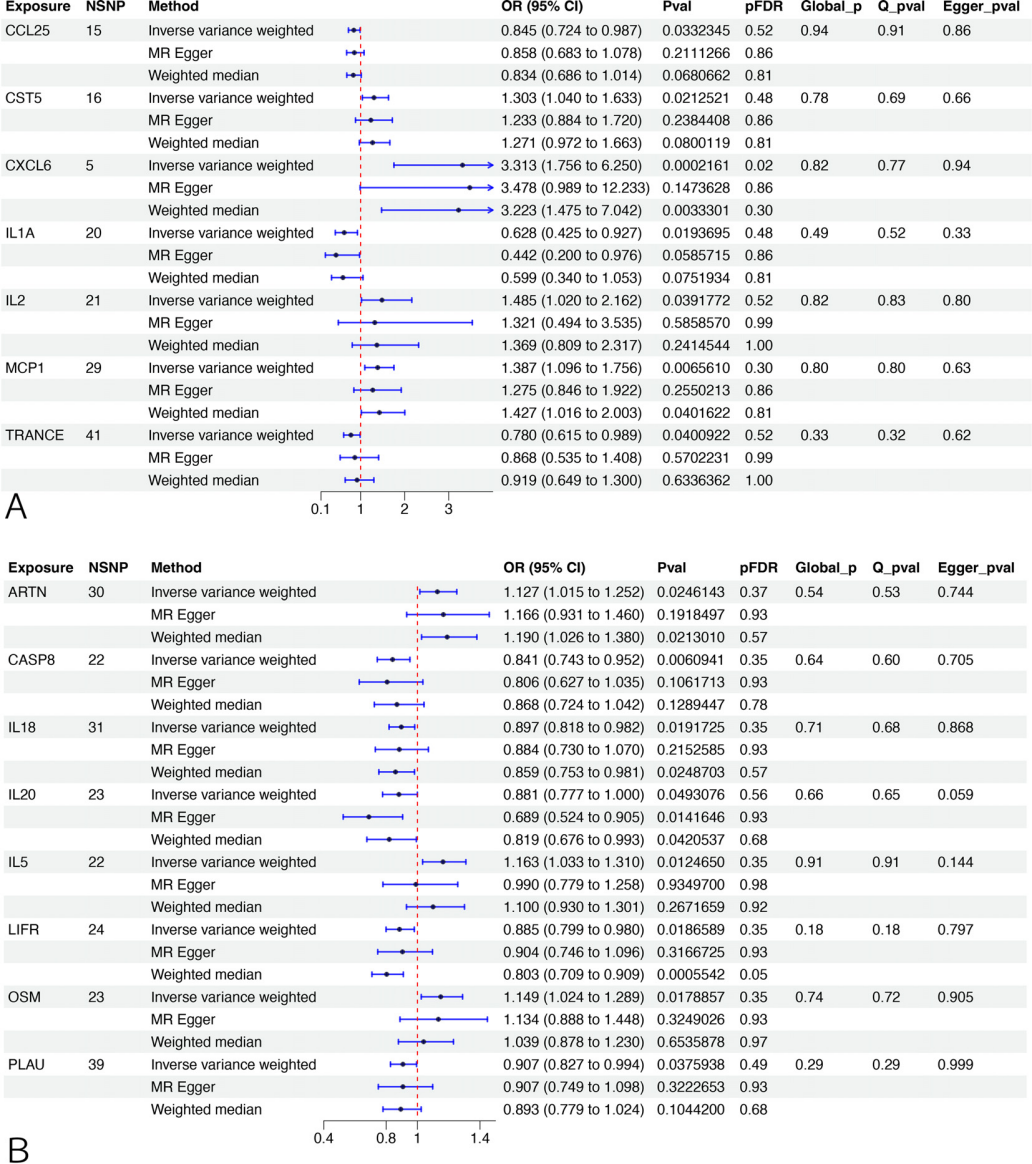
MR analysis between CIPs and infertility. **(A)** The association between CIPs trait and male infertility. **(B)** The association between CIPs trait and female infertility. NSNP, number of single-nucleotide polymorphism. OR (95%CI): odds ratio (95% confidence interval). FDR, false discovery rate. A pFDR<0.05 was deemed a significant CIP, and Pval <0.05 indicated a potential CIP. Q_pval, p value of Cochran’s Q test. Egger_pval, p value of MR-Egger regression (MR-ER) method. Global_p, p value of MR-PRESSO.

Two-sample MR and IVW analyses were performed to reveal any causal effects of CIPs on male infertility. After FDR adjustment, only C-X-C motif chemokine 6 (CXCL6) was found to have a positive relationship with male infertility (95% CI: 1.7564 to 6.2502, pFDR=0.01966) and considered a significant CIP. Another 6 CIPs were identified as having potentially significant relationships impacting male infertility, including 3 beneficial: Monocyte chemoattractant protein-1 (MCP1) (95% CI: 1.0956 to 1.7563, p=0.0066), Cystatin D (CST5) (95% CI: 1.0403 to 1.6323, p=0.0213), and Interleukin-2 (IL2) (95% CI: 1.0198 to 2.1619, p=0.0392) and 3 deleterious: Interleukin-1-alpha (IL1A) (95% CI: 0.4252 to 0.9275, p=0.0194), C-C motif chemokine 25 (CCL25) (95% CI: 0.7241 to 0.9867, p=0.0332), and TNF-related activation-induced cytokine (TRANCE) (95% CI: 0.6154 to 0.9888, p=0.0401). These results were further validated by LOO testing. Sensitivity analysis confirmed heterogeneity in the results without evidence of pleiotropy (**Figure 2**, **Supplementary Table S2**). MR-PRESSO analysis showed no horizontal pleiotropy and identified outliers (**Supplementary Table S2**), and bidirectional MR analyses did not identify significant causal relationships between male infertility and 7 positive CIPs (**Supplementary Table S3**).

### Associations between CIPs and female infertility

After FDR adjustment, no CIPs were identified as having potential casual relationships with female infertility compared to non-FDR adjusted data, which identified 8 potentially significant CIPs. Of these, 3 had a beneficial impact on female infertility: Interleukin-5 (IL5) (95% CI: 1.0332 to 1.3101, p=0.0125), Oncostatin-M (OSM) (95% CI: 1.0242 to 1.2889, p=0.0179), and Artemin (ARTN) (95% CI: 1.0155 to 1.2518, p=0.0246), and 5 had a deleterious effect: Caspase 8 (CASP8) (95% CI: 0.7427 to 0.9517, p=0.0061), Leukemia inhibitory factor receptor (LIFR) (95% CI: 0.799 to 0.9798, p=0.0187), Interleukin-18 (IL18) (95% CI: 0.8182 to 0.9823, p=0.0192), Urokinase-type plasminogen activator (PLAU) (95% CI: 0.8273 to 0.9944,p=0.0376), and Interleukin-20 (IL20) (95% CI: 0.7768 to 0.9996, p=0.0493). Sensitivity analysis confirmed heterogeneity in the results without pleiotropy (**Figure 2**, **Supplementary Table S2**). MR-PRESSO analysis showed no horizontal pleiotropy and identified outliers (**Supplementary Table S2**). Bidirectional MR analyses also did not find evidence of significant causal relationships between female infertility and the 8 significant CIPs (**Supplementary Table S3**).

### Mediation analysis

Given that CIPs may influence plasma metabolites, we used 1400 plasma metabolites from a prior study by Chen et al. (24) for mediation analysis.

**Figure 3:**
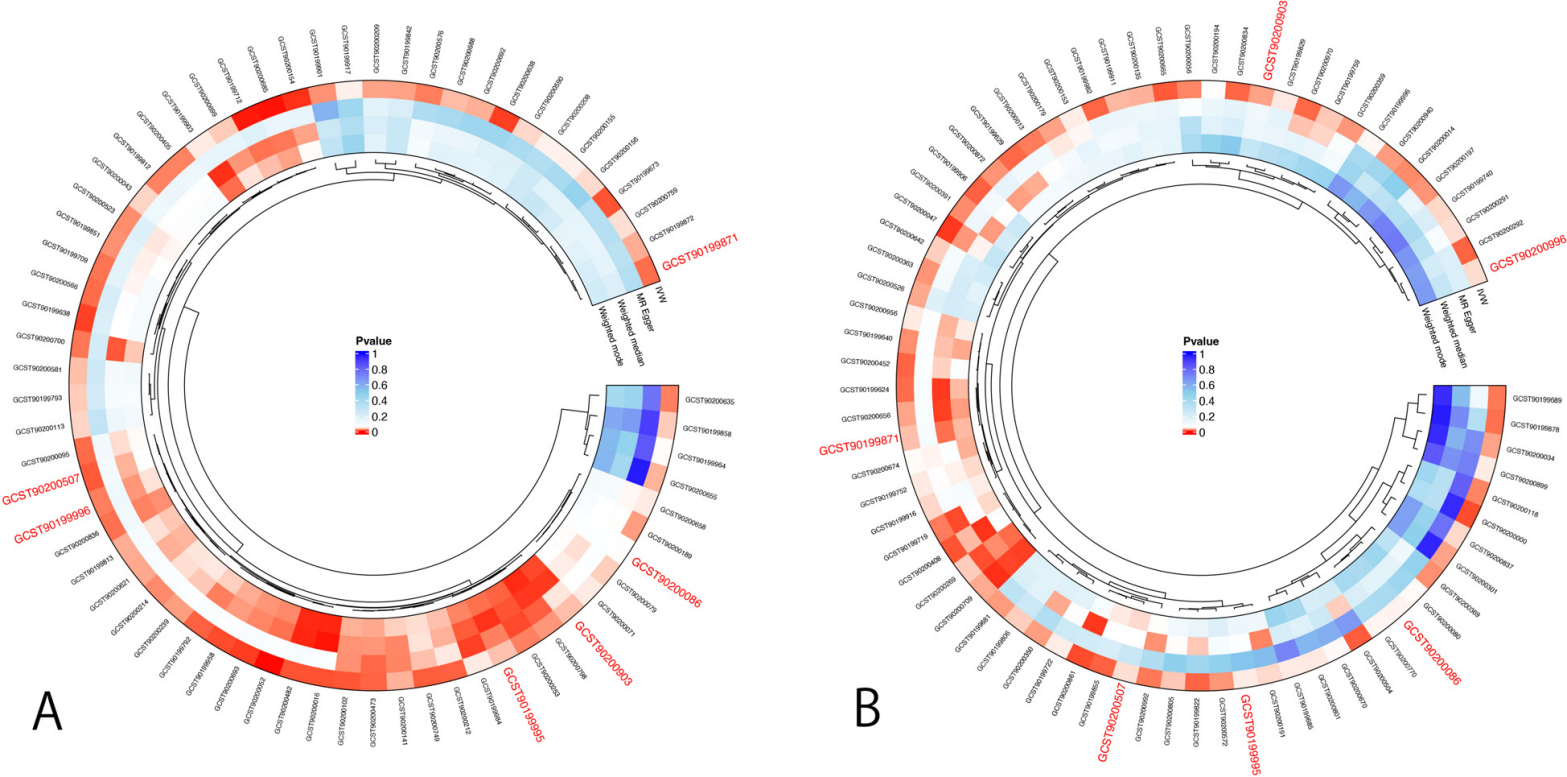
MR analysis for mediation analysis. **(A)** Positive results of IVW method for the association between CXCL6 and 1400 plasma metabolites. **(B)** Positive results of IVW method for the association between 1400 plasma metabolites and male infertility. The p-value represents significance. The smaller the p-value, the redder the color is, indicating a more significant result. Red color ID represents the GWAS code of potential mediators.

First, IVs for CXCL6 were used to evaluate the potential causal effects of CXCL6 exposure on the plasma metabolites. Using IVW analysis, we found potential causal effects of CXCL6 on 57 of the 1400 tested plasma metabolites (**Figure 3**, **Supplementary Table S4**). Next, we assessed the potential associations of these plasma metabolites with male infertility. Sixty-one plasma metabolites were identified as having causal effects on male infertility via IVW analyses (**Figure 3**, **Supplementary Table S4**). Of the total 118 plasma metabolites associated with CXCL6 or male infertility, androstenediol (3beta,17beta) monosulfate (2) levels, sphingomyelin (d18:1/20:2, d18:2/20:1, d16:1/22:2) levels, sphingomyelin (d18:1/20:1, d18:2/20:0) levels, arachidonoylcholine levels, X-12822 levels, and phosphate to glycerol ratio appeared in both groups.

We estimated the indirect effect of CXCL6 on male infertility via 6 potential mediators (**Table 2**), however, the results were not significant. Androstenediol (3beta,17beta) monosulfate (2) levels, arachidonoylcholine (AA-CHOL) levels, and phosphate to glycerol ratio had 1.4%, 1.2%, and 1.85% mediation effect, respectively. While sphingomyelin (d18:1/20:2, d18:2/20:1, d16:1/22:2) levels, sphingomyelin (d18:1/20:1, d18:2/20:0) levels and X-12822 levels indicated suppressing effects instead of any mediating effects (38). Additionally, the bidirectional MR analyses didn’t find any evidence of significant causal relationships between 6 potential mediators and CXCL6 (**Supplementary Table S5**), suggesting that CXCL6 cannot serve as a mediator as well.

**Table 2:** The mediation effect of CXCL6 on male infertility via 1400 plasma metabolites.

Exposure	Mediator	Outcome	Total effect		Direct effect A		Direct effect B		Mediation effect		p	Mediation proportion (%) (95%CI) (Suppressing effect)
			b	se	b	se	b	se	b	se		
CXCL6	Androstenediol (3beta,17beta) monosulfate (2) levels	Male Infertility	1.1979	0.3238	-0.0787	0.0321	-0.2124	0.1002	0.0167	0.0109	0.1253	1.4 (-0.39 to 3.18)
CXCL6	Sphingomyelin (d18:1/20:2, d18:2/20:1, d16:1/22:2) levels	Male Infertility	1.1979	0.3238	0.0640	0.0302	-0.1803	0.0899	-0.0115	0.0084	0.1684	-0.96 (-2.33 to 0.41)
CXCL6	Sphingomyelin (d18:1/20:1, d18:2/20:0) levels	Male Infertility	1.1979	0.3238	0.0724	0.0305	-0.1873	0.0947	-0.0136	0.0094	0.1483	-1.13 (-2.67 to 0.4)
CXCL6	Arachidonoylcholine levels	Male Infertility	1.1979	0.3238	0.0646	0.0329	0.2228	0.1121	0.0144	0.0109	0.1885	1.2 (-0.59 to 2.99)
CXCL6	X-12822 levels	Male Infertility	1.1979	0.3238	-0.0847	0.0328	0.2171	0.1066	-0.0184	0.0120	0.1262	-1.53 (-3.5 to 0.43)
CXCL6	Phosphate to glycerol ratio	Male Infertility	1.1979	0.3238	-0.0644	0.0302	-0.3449	0.1549	0.0222	0.0152	0.1432	1.85 (-0.63 to 4.33)

‘Total effect’ indicates the effect of CXCL6 on male infertility.

‘Direct effect A’ indicates the effect of CXCL6 on plasma metabolites.

‘Direct effect B’ indicates the effect of plasma metabolites on male infertility and ‘mediation effect’ indicates the effect of CXCL6 on male infertility through mediators.

Total effect, direct effect A and direct effect B were derived by IVW; mediation effect was derived by using the delta method.

All statistical tests were two-sided. P < 0.05 was considered significant.

### PheWAS and phenotype scanning analysis

PheWAS results can be interpreted as the association of genetically determined protein expression with specific diseases or traits. We first identified 7 CXCL6-associated SNPs from previous MR analyses, 4 of which successfully passed clumping and matched to 4 control SNPs each (**Supplementary Table S6**). PheWAS analyses were then performed with the UK Biobank GeneATLAS database to test each CXCL6-associated variant and control variant for association with 778 traits. A total of 6 traits were more likely to be associated with CXCL6 SNPs than with control SNPs at statistical significance (p<0.05), including neutrophil count, lymphocyte percentage, neutrophil percentage, white blood cell (leukocyte) count, monocyte count, and basophil percentage (**Supplementary Table S7**).

In phenotype scanning, CXCL6 (rs12075, rs13148728, rs597808, rs74361503) expression was found to have associations with 138 other traits (**Supplementary Table S8**), 18 of which were related to disease. We then used MR to check the causal relationship between CXCL6 and 18 related diseases, but no causal relationship was identified (**Supplementary Table S9**). Collectively, these data suggest that CXCL6 may serve as a plasma molecular biomarker for male infertility.

### Functional enrichment analysis of positively associated CIPs

**Figure 4:**
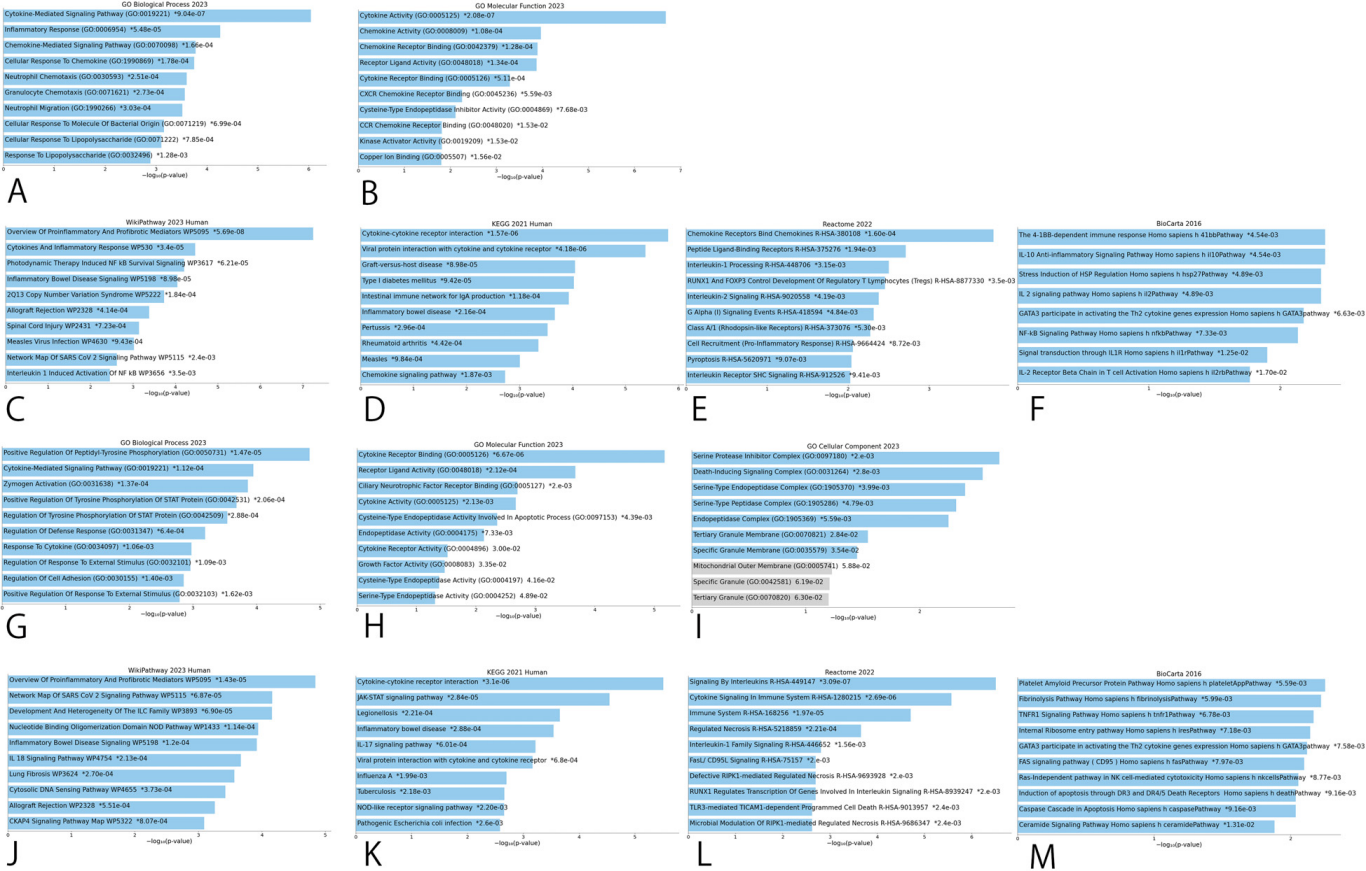
Bar graphs of GO enrichment analysis and pathway enrichment analysis for potential causal CIPs. **(A)** Top 10 terms of male infertility in BP. **(B)** Top 10 terms of male infertility in MF. **(C)** WikiPathway of male infertility. **(D)** KEGG Pathway of male infertility. **(E)** Reactome Pathway of male infertility. **(F)** BioCarta Pathway of male infertility. **(G)** Top 10 terms of female infertility in BP. **(H)** Top 10 terms of female infertility in MF. **(I)** Top 10 terms of female infertility in CC. **(J)** WikiPathway of female infertility. **(K)** KEGG Pathway of female infertility. **(L)** Reactome Pathway of female infertility. **(M)** BioCarta Pathway of female infertility. BP, Biological Process; MF, Molecular Function; CC, Cellular Component.

GO analyses in male infertility identified potential CIPs chiefly involved in cytokine pathways with effects on cytokine activity (**Figure 4**), however in female infertility, potential CIPs had effects on cytokine receptor binding, serine protease inhibitor complex, or pro-inflammatory and profibrotic mediators (**Figure 4**).

The pathways with the strongest implications for potential CIPs were captured among four global bases, including WikiPathway, KEGG, Reactome, and BioCarta. These results identified pro-inflammatory and profibrotic mediators and cytokine-cytokine receptor interaction as the strongest pathways in male and female infertility in WikiPathway and KEGG, respectively. Inflammatory Bowel Disease Signaling also appeared in 4 pathway results (**Figure 4**).

### PPI network construction

**Figure 5:**
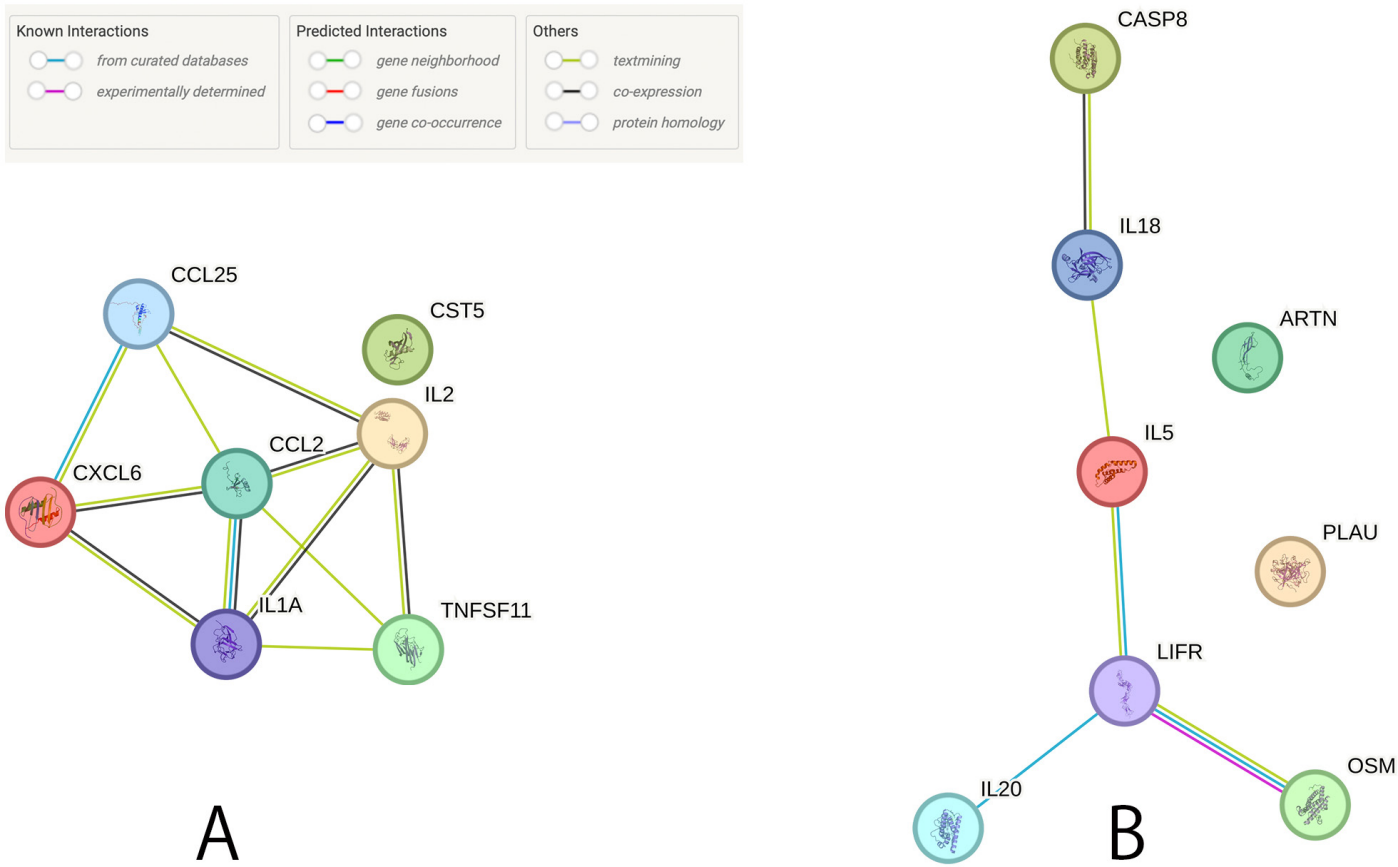
Protein-protein interaction network among the potential causal CIPs. **(A)** Relationship between the suggestive causal CIPs (P < 0.05) in male infertility. **(B)** Relationship between the suggestive causal CIPs (P < 0.05) in female infertility.

We generated a PPI network of potential CIPs in male and female infertility (**Figure 5**). Of the potential CIPs, IL2 and IL1A had the highest combined score (0.997) in male infertility, and OSM and LIFR had the highest combined score (0.999) in female infertility (**Supplementary Table S10**).

### Gene-drug interactions

We then used Enrichr to analyze the positive or negative relationships between potential CIPs infertility and identify drugs that may modulate these CIPs using the DSigDB database (**Tables 3**, **4**). For positively related CIPs indicated that manganese and pioglitazone are the top drug candidates for male and female infertility, respectively. With respect to negatively related CIPs, leupeptin and histamine emerged as the top drug candidates for treating male and female infertility, respectively.

**Table 3:** Suggested top 10 drugs for male infertility.

Drugs predict for male infertility positive related CIPs						Drugs predict for male infertility negative related CIPs					
Term	P-value	Adjusted P-value	Odds Ratio	Combined Score	Genes	Term	P-value	Adjusted P-value	Odds Ratio	Combined Score	Genes
MANGANESE CTD 00006240	0.0007	0.0476	93.7678	685.5760	CXCL6; IL2	Leupeptin CTD 00001574	0.0016	0.0251	999.3500	6403.3348	IL1A
BETAMETHASONE VALERATE CTD 00005505	0.0022	0.0476	666.2000	4077.1879	IL2	6401-97-4 CTD 00000925	0.0016	0.0251	999.3500	6403.3348	IL1A
NICKEL CHLORIDE CTD 00001064	0.0023	0.0476	49.3678	299.4843	CXCL6; IL2	BENZOYL PEROXIDE CTD 00005495	0.0016	0.0251	999.3500	6403.3348	IL1A
buprenorphine CTD 00005539	0.0024	0.0476	605.6061	3653.6995	IL2	urethane CTD 00006966	0.0021	0.0251	768.6154	4739.6562	IL1A
Modrasone CTD 00001031	0.0024	0.0476	605.6061	3653.6995	IL2	POVPC CTD 00003737	0.0022	0.0251	713.6786	4351.6858	IL1A
methanol CTD 00005338	0.0028	0.0476	512.3846	3012.3745	IL2	Estrone sulfate CTD 00000891	0.0024	0.0251	666.0667	4018.4162	IL1A
urethane CTD 00006966	0.0028	0.0476	512.3846	3012.3745	IL2	sodium chloride CTD 00006751	0.0024	0.0251	666.0667	4018.4162	IL1A
propanil CTD 00004257	0.0030	0.0476	475.7619	2764.2762	IL2	nefazodone CTD 00002219	0.0024	0.0251	666.0667	4018.4162	IL1A
KN-62 CTD 00002580	0.0032	0.0476	444.0222	2551.2387	IL2	AC1L1WKQ CTD 00007050	0.0027	0.0251	587.6471	3476.1500	IL1A
Phorbol 12-myristate 13-acetate CTD 00006852	0.0034	0.0476	40.5717	230.8336	CXCL6; IL2	Nebivolol CTD 00002249	0.0027	0.0251	587.6471	3476.1500	IL1A

**Table 4:** Suggested top 10 drugs for female infertility.

Drugs predict for female infertility positive related CIPs						Drugs predict for female infertility negative related CIPs					
Term	P-value	Adjusted P-value	Odds Ratio	Combined Score	Genes	Term	P-value	Adjusted P-value	Odds Ratio	Combined Score	Genes
pioglitazone PC3 DOWN	1.1527E-05	0.0022	665.8333	7571.0827	PLAU; IL18	histamine BOSS	0.0002	0.0163	222.6854	1855.8501	IL5; ARTN
(-)-Epigallocatechin gallate BOSS	1.1560E-05	0.0022	142.0048	1614.3065	CASP8; PLAU; IL18	CORTICOSTERONE BOSS	0.0002	0.0163	222.6854	1855.8501	IL5; ARTN
gemcitabine CTD 00002382	1.5740E-05	0.0022	127.7780	1413.1374	CASP8; PLAU; LIFR	PIPERONYL BUTOXIDE BOSS	0.0003	0.0163	206.3021	1688.4212	IL5; ARTN
15442-64-5 CTD 00000915	1.8851E-05	0.0022	512.0256	5570.3046	CASP8; IL18	BETAMETHASONE VALERATE CTD 00005505	0.0016	0.0313	999.3500	6403.3348	IL5
Honokiol CTD 00000234	2.0245E-05	0.0022	493.0370	5328.5455	CASP8; IL18	Beclomethasone 17-monopropionate CTD 00002649	0.0016	0.0313	999.3500	6403.3348	IL5
Destruxin B CTD 00001859	3.1393E-05	0.0022	391.3922	4058.3164	CASP8; PLAU	Modrasone CTD 00001031	0.0018	0.0313	908.4545	5741.9213	IL5
Luronit CTD 00006106	3.1393E-05	0.0022	391.3922	4058.3164	CASP8; PLAU	Dimaprit CTD 00007150	0.0022	0.0313	713.6786	4351.6858	IL5
2-Naphthoxyacetic acid BOSS	3.5024E-05	0.0022	369.6111	3792.0209	CASP8; PLAU	Desloratadine CTD 00003720	0.0024	0.0313	666.0667	4018.4162	IL5
mitomycin C CTD 00007136	3.5349E-05	0.0022	96.8361	992.5936	CASP8; PLAU; IL18	terbinafine CTD 00001890	0.0024	0.0313	666.0667	4018.4162	IL5
AMILORIDE CTD 00005369	0.0001	0.0030	295.5556	2905.3408	CASP8; PLAU	HEXACHLOROBENZENE CTD 00006091	0.0025	0.0313	624.4063	3729.2533	IL5
Melitten CTD 00006261	0.0001	0.0035	260.7059	2499.6356	CASP8; IL18	leukotriene D4 CTD 00007224	0.0025	0.0313	624.4063	3729.2533	IL5

## Discussion

Infertility is a complex problem affecting both males and females through varied processes. Male infertility etiologies include genetic mutations (39), autoimmune responses (40), oxidative stress (41), and inflammatory indicators (42). Female infertility has also been linked to oxidative stress (43) and inflammatory mechanisms (44). Here, we use two-sample Mendelian Randomization to investigate infertility-associated CIPs and plasma metabolites linked to infertility. We identified CXCL6 as a promising therapeutic target and possible plasma biomarker for male infertility. Using the infertility-associated CIPs, we also explored potential infertility mechanisms using enrichment analyses, Protein-Protein Interaction (PPI), and Gene-drug interaction studies. We found some potential drugs like leupeptin or pioglitazone which are already used for some diseases, thereby advancing the understanding of both male and female infertility.

CXCL6 is a chemokine involved in neutrophil trafficking and activation that has been implicated in the pathogenesis of several diseases and has been shown to promote cancer cell metastasis, fibrosis, and inflammation (45–47). Moreover, studies have suggested that CXCL6 can be regulated by specific cell signaling pathways, such as the HIF-1α pathway in hepatocellular carcinoma and the IL4 pathway through the JAK-STAT mechanism in atopic dermatitis (48, 49). The diverse roles of CXCL6 in various diseases and its involvement in cellular processes and the tumor microenvironment make it a promising drug target for therapeutic intervention in multiple pathological conditions. Our findings align with existing research identifying CXCL6 as a potential plasma biomarker in many conditions (50–52). Significantly, our phenotype analysis and PheWAS results indicate that CXCL6 is not associated with other diseases. This lack of association with additional conditions enhances its suitability as a specific plasma biomarker for male infertility, offering a more targeted and potentially accurate diagnostic tool in this context. Our findings propose that CXCL6 not only warrants in-depth study but also presents potential as both a drug target and a plasma molecular biomarker for male infertility.

In our study, we analyzed potentially causal links between 1,400 plasma metabolites and male infertility using the Inverse Variance Weighted (IVW) methodology. This approach led to the identification of 66 metabolites positively correlated with male infertility. Notably, we observed that levels of androstenediol (3beta,17beta) monosulfate (2), AA-CHOL, and the phosphate-to-glycerol ratio were associated with both CXCL6 and male infertility. Androstenediol (3beta,17beta) monosulfate (2) is a metabolite involved in various physiological functions. Matsui et al. (53) noted its role in testosterone sulfate metabolism in rats, while other studies have identified it as a precursor in testosterone synthesis from DHEA (54). Additionally, the anabolic steroid 19-nor-4-androstenediol-3 beta and one of its derivatives 17 beta-diol (3 beta,19-NA), have been hypothesized to increase muscle and bone mass with minimal prostate stimulation (55). AA-CHOL acts as an endogenous modulator of acetylcholine signaling, found in atherosclerotic plaques and the arterial intima (56), and is known to regulate cholinesterases (57). Acetylcholine is involved in penile erection and relaxation as well as the central control of ejaculation. Acetylcholinesterase is found to plays role in the pathogenesis of male infertility via modulating inflammatory pathways (58). These data suggested that AA-CHOL might affect male fertility via modulating acetylcholine and acetylcholinesterase activities. Furthermore, phosphate is crucial for male fertility and affects sperm motility and function, as well as semen liquefaction (59, 60). However, its role in correcting infertility is unclear, as simple dietary supplementation cannot reverse infertility in mice (61). Glycerol plays a critical role in lipid metabolism and sperm motility but has both beneficial and harmful effects on sperm health and viability (62–64). Exactly how the phosphate-to-glycerol ratio affects male infertility merits further research.

Our enrichment analysis underscored the importance of cytokine-related pathways in infertility, aligning with existing research that implicates a deleterious role of cytokines in both male and female infertility. Cytokines are signaling molecules that play crucial roles in testicular function and male infertility. Cytokines like interleukin 1β (IL-1β), IL-6, IL-10, IL-18, tumor necrosis factor α (TNF-α), interferon g (IFN-g), and transforming growth factor β1 (TGF-β1) are notably elevated in idiopathically infertile males and those with varicocele (65, 66). Attia et al. (67) also highlighted the significant role of pro-inflammatory cytokines and microRNAs in male infertility. Cytokines in the male gonad primarily originate from testicular macrophages, with Leydig and Sertoli cells also contributing. Additionally, cytokines, such as IL2 and IL1A had the highest combined score in the PPI network associated with male infertility. IL-2 plays a crucial role in T-cell mediated immune responses. IL-2 levels in seminal plasma are found to be related to sperm count, motility, and morphology, and may be a potential marker in male infertility (68). IL-2 concentrations are closely associated with Testosterone hormone levels in COVID-19 infertile patients (69). IL-1 is primarily secreted by macrophages and monocytes in reaction to foreign antigens and inflammatory stimuli. A previous study has demonstrated that IL1A is involved in testicular paracrine/autocrine regulation, potentially influencing spermatogenesis, spermiogenesis, and male fertility (70). In female infertility, it has been shown that cytokine profiles are differentially expressed in the peritoneal fluid of patients with and without endometriosis being evaluated for infertility (71). Notably, the levels of CXCL6 are influenced by other cytokines and reproductive hormones, further supporting its function in both normal and abnormal human reproduction (72). A deeper understanding of cytokines’ roles in infertility could lead to the development of novel therapeutic approaches for those facing conception challenges.

Our gene-drug interaction analysis for positively related CIPs indicated that manganese and pioglitazone are the top drug candidates for male and female infertility, respectively. Manganese is necessary for metabolism, growth, and reproduction (73), but excess levels have been linked to male infertility (74) via mechanisms that include increased spontaneous abortion rates in partners, impotence, and infertility (75). Pioglitazone already shows potential in treating female infertility, particularly in polycystic ovary syndrome (PCOS). Nagao et al. (76) found that pioglitazone can suppress excessive ovarian follicule development in mice, suggesting a mechanism explaining its impact on reproductive health. With respect to negatively related CIPs, leupeptin and histamine emerged as the top drug candidates for treating male and female infertility, respectively. Leupeptin is a protease inhibitor used in the treatment of AIDS, hepatitis, pancreatitis, and cancer (77) with high antiviral activity against influenza and coronavirus, notably binding strongly with the TMPRSS2 protease in COVID-19 (78). Leupeptin also acts as an autophagy-blocking agent, which is implicated in its mechanism of action in the treatment of HIV-1-associated neurological disorders (79), yet a role for it in the treatment of male infertility is unknown. Histamine has a regulatory effect on the female reproductive system and is known to influence the electrophysiology of the human oviduct, affecting fertility (80, 81). Together, these two molecules merit further study in the treatment of female infertility.

Limitations of this study included the analysis of disease-causing effects identified in different studies, and inconsistent measurements across studies might have introduced biases in results. Additionally, only one target CXCL6 passed our initial FDR correction, so we performed other analyses on potential associated targets. Moreover, the CXCL6 detection results was not validated in clinical male infertility patients, and further studies are required to confirm the diagnostic value of CXCL6 and its practical applicability as a biomarker in male infertility. Furthermore, the study population and datasets used here primarily comprise patients of European ancestry, which may limit applicability with respect to other racial and ethnic groups. Therefore, caution is needed when generalizing our results to more diverse global populations. Further research in ethnically diverse cohorts is essential to validate our results and to assess whether similar genetic associations hold true across different racial and ethnic groups. Lastly, we did not analyze the correlation between CXCL6 and ART outcomes. Moreover studies are necessary to explore this relationship and its potential role in reproductive success.

## Conclusions

Using several methods of genetic analyses and Mendelian randomization, we found that circulating CXCL6 may be causally associated with male infertility and have applications as a potential drug target or plasma molecular biomarker in male infertility. Cytokine-related pathways were also highly associated with the development and progression of infertility, and gene-drug interaction analysis suggested pioglitazone may help treat female infertility. Further studies are warranted to explore the potential of CXCL6 as a biomarker for early screening and diagnosis of male infertility, evaluate its role in therapeutic development, and assess the clinical feasibility of related drugs such as pioglitazone in improving fertility outcomes of patients struggling with infertility.

## Data Availability

The original contributions presented in the study are included in the article/**Supplementary Material**. Further inquiries can be directed to the corresponding author.
